# The causal effect of delivery volume on severe maternal morbidity: an instrumental variable analysis in Sichuan, China

**DOI:** 10.1136/bmjgh-2022-008428

**Published:** 2022-05-10

**Authors:** Nan Chen, Jay Pan

**Affiliations:** 1 HEOA Group, West China School of Public Health and West China Fourth Hospital, Sichuan University, Chengdu, People's Republic of China; 2 Institute for Healthy Cities and West China Research Center for Rural Health Development, Sichuan University, Chengdu, People's Republic of China

**Keywords:** Obstetrics, Maternal health, Health policy

## Abstract

**Objective:**

Findings regarding the association between delivery volume and maternal health outcomes are mixed, most of which explored their correlation. This study aims to demonstrate the causal effect of delivery volume on severe maternal morbidity (SMM) in China.

**Methods:**

We analysed all women giving birth in the densely populated Sichuan province with 83 million residents in China, during the fourth quarters of each of 4 years (from 2016 to 2019). The routinely collected discharge data, the health institutional annual report data and road network data were used for analysis. The maternal health outcome was measured by SMM. Instrumental variable (IV) methods were applied for estimation, while the surrounding average number of delivery cases per institution was used as the instrument.

**Results:**

The study included 4545 institution-years of data from 1456 distinct institutions with delivery services, reflecting 810 049 associated delivery cases. The average SMM rate was approximately 33.08 per 1000 deliveries during 2016 and 2019. More than 86% of delivery services were provided by a third of the institutions with the highest delivery volume (≥143 delivery cases quarterly). In contrast, less than 2% of delivery services were offered by a third of the institutions with the lowest delivery volume (<19 delivery cases quarterly). After adjusting the confounders in the IV-logistic models, the average marginal effect of per 1000 cases in delivery volume was −0.162 (95% CI −0.169 to –0.155), while the adjusted OR of delivery volume was 0.005 (95% CI 0.004 to 0.006).

**Conclusion:**

Increased delivery volume has great potential to improve maternal health outcomes, while the centralisation of delivery services might facilitate maternal health promotion in China. Our study also provides implications for other developing countries confronted with similar challenges to China.

**WHAT IS ALREADY KNOWN ON THIS TOPIC**

- Findings of the association between delivery volume and maternal health outcomes are mixed.
- Limited studies explore the causal effect of delivery volume on adverse maternal health outcomes.

**WHAT THIS STUDY ADDS**

- The instrumental variable method was first applied to identify the causal effect of delivery volume on severe maternal morbidity (SMM).
- Information of 810 049 delivery-related discharge records from China during 2016 and 2019 were used for the empirical analysis.
- An increase of 1000 deliveries would reduce the 16.2% rate and get a 0.005 OR of SMM.

**HOW THIS STUDY MIGHT AFFECT RESEARCH, PRACTICE AND/OR POLICY**

- This study provides evidence for the causal effect of delivery volume on SMM in China.
- Centralisation of obstetric delivery services would be an option to improve maternal health for developing countries facing similar challenges.

## Introduction

Whether or not the centralisation of health services should be adopted as a strategy to facilitate population health promotion is a study focus both for policy-making and research purposes.^1–6^ Centralisation policy for resource allocation also is described as ‘concentration’ or ‘regionalization’.^7 8^ Volume-outcome is the basis for promoting centralisation policy.^9^ For many surgeries, the volume has been demonstrated to be positively associated with health outcomes because of learning effects or economies of scale.^2 9 10^ In the obstetric field, scholars have conducted several studies to investigate the relationship between delivery volume and maternal health outcomes, which, however, provided mixed findings. While some studies indicated that delivery volume is positively correlated with maternal health outcomes,^11–15^ other studies reported the absence of a statistically significant correlation between them.^16–19^

It is challenging to explore the relationship between delivery volume and maternal health outcomes by experimental research. Because considering the multiple stakeholders, the researchers have to not only obtain the permission of administrators but also seek support and cooperation from medical institutions and pregnant women. Existing studies had to merely rely on observational data for analysis. It should be noticed that these studies generally adopted empirical analytical strategies to investigate such association. Due to the presence of confounding factors, such association identified might be a biased estimate. In other words, confounding factors or endogenous problems come from two aspects. First, the two study objects are in a simultaneous relationship where delivery volume and obstetric health outcomes affect each other. Specifically, while the delivery volume would affect maternal health outcomes, such outcomes would also affect the choice of mothers in seeking hospital services, thus further affecting the delivery volume. Second, unobservable heterogeneous confounding factors might be induced by patients in the association analyses. For instance, high-risk women might prefer to seek hospital services from healthcare institutions with better health outcomes, while they are more likely to have adverse outcomes.

To bridge the gap in the current literature, the instrumental variable (IV) method was adopted for the first time to identify the causal effects of delivery volume on maternal health outcomes. The IV method is a compelling analysis approach to explore causal effects based on observed data.^20^ It usually uses one or more exogenous IVs that are related to the critical exposure variable but not directly related to the outcome variable, to identify the impacts of the exposure variable on the explained variable, which could lead to a consistent estimation. This study selected the surrounding average number of delivery cases per institution as the IV. The selection was based on the assumption that the number of delivery cases in the surrounding area of a specific hospital is positively related to its actual delivery volume without directly affecting the maternal health outcomes produced by that specific hospital. The results of this study will provide more substantial evidence on the causal effect. We tried to demonstrate whether the rising delivery volume could improve maternal health outcome by applying the IV method in this paper.

This study used maternal information from a populated province with 83 million residents during the fourth quarters of each of 4 years (through 2016 to 2019) in China to conduct the analysis. To date, existing evidence on volume-outcome was provided by high-income countries.^11–19 21^ However, low-income and middle-income countries are quite different from high-income countries in terms of economic development status, educational levels, sanitary conditions and other factors that may affect the volume-outcome relationship. As the world’s largest developing country, the results from China have great potential to inform health-related decision-making procedures among other low-income and middle-income countries confronted with similar issues.

## Methods

### Study area and data source

Sichuan province is located in south-western China with a 486 000 km^2^ area and 83.67 million residents. As the province has many similarities with the nationwide situation of China in terms of the geographical, demographical and economic distribution characteristics, the findings based on this study area were believed to have relatively good external validity.^22^ In the east of China, the main geomorphological features are plains and hills with nearly 41% of the country’s population and a high level of economic development. In contrast, the western region of China is dominated by plateaus and mountains with a relatively sparse population and a low-level economic development. Similarly, the east of Sichuan province is dominated by plains and hills, with a dense population and rapid economic growth, while the west of Sichuan is dominated by mountainous areas, with a sparse population and lagging economic development. These gaps between different regions lead to significant disparities in the development level of medical and health services, including obstetric services. The quarterly delivery volume ranges from 1 to 5664 in our database, which provides the possibility of applying the IV estimation method.

All the discharge data regarding inpatients discharged during the fourth quarters between 2016 and 2019 (1 October to 31 December 2016–2019) were included for analysis. The discharge data contained a list of essential demographical characteristics, the disease diagnoses, the conditions and dates for admission and discharge, the surgery and operation procedures, the mode of discharge, and the expenditure information for every single case.^23^ The health institution annual report data between 2016 and 2019 were adopted to describe institutional characteristics, including the address, ownership types, hospital level and the number of beds in each health institution.^24^ These two data sets were extracted from the Sichuan Health Statistics Data Collection and Decision Support System. Public statistical yearbook data between 2016 and 2019 were used to describe region-specific characteristics. The latest version of road network data was retrieved from the National Catalogue Service for Geographic Information system. The geographical coordinates of health institutions and patients were obtained based on their addresses by using the geocoding Application Programming Interface of Baidu Map, a web-based map on China’s internet frequently accessed by Chinese residents.^25^ The travel time between patients and healthcare institutions or between two different institutions was calculated through ArcGIS V.10.5 combined with the coordinate information and the road network data.

**Figure 1:**
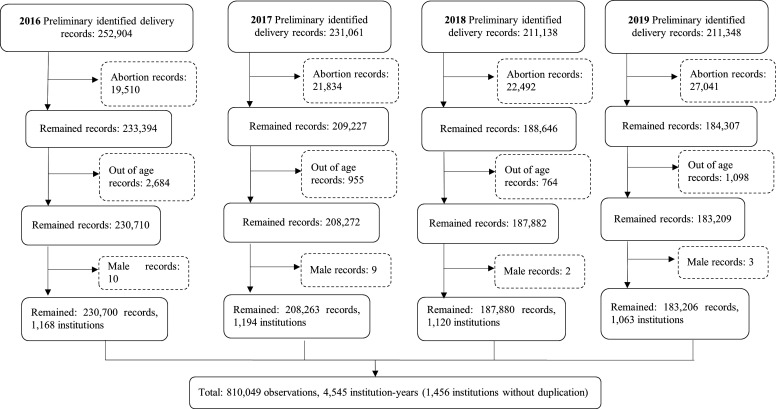
Data screening flow diagram.

The data screening was performed via the following steps. First, all the records potentially related to delivery were identified. The discharge data collected in the last quarters between 2016 and 2019 in Sichuan province were screened for extracting delivery records. The number of preliminary identified delivery-related records was 906451. Then the following exclusion criteria were adopted to ensure the data validity: (1) Abortion records; (2) Age not between 15 years and 49 years old; (3) Male records. As the result of data screening, a total of 810 049 records were retained (figure 1).

The quality of the discharge data has always been the focus of China’s health administrative department. With the gradual promotion of diagnosis-related groups in China, the coding quality of the discharge data is increasingly important. Since many indicators of performance evaluation of health institutions are calculated based on discharge data, institutions pay more and more attention to the quality of data.

The quality of data was generally acceptable. According to the bulletin, the average admission cases in Sichuan province within one quarter was approximately 4.56–4.95 million, and the infacility delivery rate was 99.32%–99.77%.^26^ Our database contained 4.57–4.97 million discharge records, indicating that our data had great potential to reflect the overall situation of inpatient services delivered by all healthcare institutions across Sichuan province.

Based on the number of neonatal births recorded in the health statistical yearbook, the average amount of infacility births within one quarter was predicted to be 243 894–255 328.^26^ The total number of deliveries within one quarter in our database ranged from 186 353 to 210 910 (including multiple births). Despite the fluctuations in the number of delivery cases during different seasons and the discrepancies embedded in coding quality, about 78%–85% of cases out of total maternal deliveries were successfully identified from the discharge database.

### Variables

We applied severe maternal morbidity (SMM) as the maternal health outcome indicator. SMM is one of the most commonly adopted obstetric outcome indicators in many countries.^27 28^ It is also described as ‘maternal near miss’ or ‘near miss morbidity’. The WHO has defined SMM as ‘a woman who nearly died but survived a complication’.^27 28^ The Centers for Disease Control and Prevention (CDC) in the USA recommended SMM as a monitoring quality indicator of maternal care.^29 30^ In recent years, some researchers in China started to promote SMM as a maternal outcome indicator.^28 31^ In this study, we adapted the SMM definition previously proposed in the USA on China’s hospital discharge data (online supplemental table 1). The International Classification of Diseases (ICD) diagnosis and procedure codes in the discharge data were employed to identify SMM cases, which contained 21 indicators. (In the continuum of SMM, maternal death is the end event that is worse than SMM. We rerun all the models with considering both SMM and death as the health outcomes. Because of the very low maternal mortality, the results showed little difference (the difference was in the fourth decimal place). Due to the space limitation, we didn’t present them in this paper.)^30^

10.1136/bmjgh-2022-008428.supp1 Supplementary data

The exposure variable was the delivery volume, defined as the number of deliveries in a quarter within each institution. Delivery volume variables were added into the models as continuous variables, and were treated as categorical variables in the descriptive analysis. The institutions were divided into three groups according to the tertiles, which was the most commonly used classification method based on the literature.^11–13 18^ As a result, the low-volume institutions had fewer than 19 deliveries per quarter, the medium-volume institutions had between 19 and 142 deliveries per quarter, while the high-volume institutions had 143 or more deliveries per quarter.

Other potential confounders including patients’ characteristics, institutional characteristics and regional characteristics were considered. The patients’ characteristics included demographical variables and socioeconomic variables such as maternal age, minority, marital status, living in rural/urban area and health insurance type. There were three types of social health insurance programmes in China during the study period:^32^ Urban Employment Basic Medical Insurance (UEBMI); Urban Residents Basic Medical Insurance (URBMI); New Cooperative Medical Scheme (NCMS). Types of social health insurance were related to socioeconomic status and available medical resources for mothers. UEBMI was for employees who have a job. URBMI was for unemployed people living in cities and towns. NCMS was for people living in rural areas. The risk level of delivery was a significant confounder for the relationship between delivery volume and SMM.^11 21^ Difference in delivery risk levels could partially reflect the heterogeneity of patients and provide evidence to inform endogenous problems. We classified deliveries into high-risk and low-risk groups based on the risk factor list. High-risk delivery contained at least one risk factor in the code list, which combined the risk factor codes in the Society for Maternal-Fetal Medicine and from the Chinese experts’ consensus^33 34^ (online supplemental table 1). In addition to risk groups, the admission source might partially reflect the delivery risk and was therefore adjusted as a confounder. The institutional characteristics included the hospital level, ownership types, number of beds, number of beds for Obstetrics and Gynaecology (OG). The regional characteristics included the gross domestic product per capita (GDP per capita) and urbanisation rate. As this study did not intend to investigate the time trends of causal effects, the time variable (year) entered the models as a dummy variable.

### Statistical analysis

In the descriptive analysis, counts and proportions were used for categorical variables, and χ^2^ tests were used for testing the differences between groups. For continuous variables, median and IQRs were used for descriptive statistics, and Kruskal-Wallis rank-sum tests were adopted to test the differences between groups.

To avoid collinearity problems, we used the variance inflation factor (VIF) to measure multicollinearity. As the independent variable SMM was a dichotomous variable, the linear probability models and traditional logistic regression models were applied. Due to limited dependent variables, the coefficient estimations of the linear model and non-linear model were very close, and both methods produced robust outcomes.^35^

The linear probability and logistic regression models could measure the association between delivery volume and SMM. In equation (1), SMM=1 indicated the occurrence of SMM. The delivery volume was indicated by V , and L were the confounders, including individual characteristics and institutional characteristics. Pr(SMM=1|V,L) was the probability of SMM conditional on exposure variable V and confounders L . The parameter of our interest was $\beta_{1}$ , which showed the relationship between delivery volume and SMM. In the linear probability model, the link function *G* was the identity link. In the logistic regression model, the link function *G* was the logit link.

(1) $$Pr\left(SMM\text{=1}|V,L\right)=G\left(\beta_{0}+\beta_{1}V+L\delta \right)$$

There were two endogeneity problems might lead to a biased estimation: the reverse causality and the unobserved patient heterogeneity. The reverse causality indicated the exchangeable direction of the causal effect. On the one hand, learning effects and economies of scale support the causal effect from delivery volume to SMM.^9 36^ On the other hand, a better outcome might lead to a good reputation with more attractiveness. Women can freely choose the delivery institution in China, resulting in a better reputation that can attract more patients. The reverse causality results in a downward bias to the coefficient of delivery volume.

The omission of unobserved patient heterogeneity might also cause the endogeneity of delivery volume. Patient characteristics could influence their choice of institution. For example, university hospitals often receive patients with more severe comorbidities.^36^ The women with worse health conditions who were more likely to have adverse outcomes may have a stronger desire to choose a better healthcare institution for deliveries. Even though we tried to adjust the delivery risk by controlling the risk groups variable, the binary risk groups were too crude to reflect the delivery risk heterogeneity among mothers in an accurate manner. Omission of patient heterogeneity might lead to an upward bias of the coefficient of delivery volume.

IV estimation was a tool for solving endogeneity problems. We chose the average number of delivery cases in the surrounding region of a specific delivery institution as the instrument variable. We assumed that the actual delivery volume for a specific institution would increase if more delivery mothers live in the area around this institution, and would decrease if other institutions in the surrounding area also provide delivery services. The ratio combined the information from both sides. Similar variables have been used as an instrument for hip fractures and coronary artery bypass graft studies.^9 36^ The searching area was limited to 2 hours’ driving distance based on the geographical accessibility target proposed by a *Lancet* commission on global surgery.^37^

This instrument variable was assumed to meet three conditions. First, it was associated with delivery volume and would only affect health outcomes through delivery volume. Distance and convenience were two influential factors for the selection of delivery institutions.^38^ In general, patients preferred to choose closer health institutions for convenience. Second, the residence of mothers could be considered as exogenous to maternal outcomes, for it should have no direct influence on the quality of health services. It is unlikely for most mothers to choose where to live just depending on the quality of obstetric delivery services. Although this condition hold in most cases, some confounder might still challenge the exclusion restriction assumption, such as the regional development level, socioeconomic status and financial situation of the mother, and the delivery risk level. The more developed regions may have a more density population and higher quality medical resources, which leads to a link between delivery volume and health outcomes. Mothers with higher socioeconomic status and better financial situation are more likely to live in areas with high-quality resources. Similarly, higher delivery risk level mothers are more motivated to live near high-quality institutions. Considering these confounding effects, we added regional characteristics, institutional characteristics and individual characteristics into the models to meet the exclusion restriction. This approach has also been used in other studies.^9 39^

It is a more rational assumption that the instrument met the second condition after controlling the potential confounders. Third, the instrument was monotonous. It was unlikely that an increased number of potential patients or a reduced number of potential institutions would lower delivery volume. Under the monotonicity condition, only a local average causal effect was explored. The IV estimation measures the causal effect for institutions obeying the monotonicity hypothesis. The estimated effect was ‘compliers average causal effect (CACE)’.

The two-stage estimation technique in the IV-logistic models was used for estimating. The first stage model was as follows:

(2) $$E\left[V|L,Z\right]=\alpha_{0}+\alpha_{1}Z+L\lambda$$

where V was the delivery volume, Z was the IV, L indicated the observed confounders. The fitted values of delivery volume were used in the following second-stage model:

(3) $$Pr(SMM=1|\hat{V},L)=G(\gamma_{0}+\gamma_{1}\hat{E}[V|L,Z]+L\eta )$$

where $\gamma_{1}$ measured the causal effect of delivery volume on SMM. Link function *G* was identity link and estimated by the ordinary two-stage least squares (TSLS) estimation approach. Link function *G* was a logit link in the IV-logistic model and was estimated by the two-stage estimation approach. In the IV-logistic model, we applied the ‘sandwich formula’ to the whole equation system to get the SE through the R package named ‘ivtools’.^39^ And the average marginal effect (AME) of delivery volume was obtained by the R package named ‘margins’. The endogeneity test was applied to test the endogeneity. *F* test of the weak instrument was applied for assessing instrument validity. All the analyses were performed with R V.4.1.1 and ArcGIS V.10.5.

Sensitivity analyses were applied for robustness testing. First, the 2 hours’ driving distance was replaced by the 1 hour driving distance to check the impact of the searching area. Second, the outcome variable SMM was recalculated with the exclusion of receiving blood products transfusion due to the controversial definition of SMM, which indicated that blood transfusion should be considered as a life-saving technique instead of an indicator reflective of SMM.^27^ Third, the institutions with less than 10 deliveries were excluded for testing the impact of extreme values.

### Patient and public involvement

Both administrative data and public data were used in this study. No patient was recruited or involved in the research. Patients were not invited to contribute to the designing, writing or editing process throughout the research.

## Results

### Descriptive analysis

A number of 4545 institution-years of data from 1456 distinct institutions were included in the analysis. Among institutions, 83.34% (3788 institution-years) were public; 14.53 (660 institution-years) were tertiary, 31.64% (1438 institution-years) were secondary, 53.84% (2447 institution-years) were primary or ungraded; 43.15% (1961 institution-years) were located in urban areas.

A total of 810 049 delivery cases were included in the analysis. The overall SMM rates fluctuated with a reduction of 5.4% from 2016 to 2019 (table 1). The overall SMM rate fluctuated up and down during the 4 years and remained below 4%. The indicator of blood products transfusion detected the largest number of SMM cases, as reported by previous studies from the USA.^30^ The rates of blood products transfusion reduced slightly during the 4 years. In contrast, the rates of severe anaesthesia complications, air and thrombotic embolism, adult respiratory distress syndrome, sepsis indicators, and ventilation significantly increased during this period, while acute renal failure and amniotic fluid embolism significantly decreased.

**Table 1:** Rates in SMM indicators per 1000 deliveries in Sichuan province of China between 2016 and 2019

SMM indicators	2016	2017	2018	2019	Rate between 2016 and 2019
Overall SMM rate	35.80	28.05	34.57	33.86	33.08
1. Acute myocardial infarction	0.004	0.000	0.005	0.006	0.004
2. Aneurysm	0.000	0.005	0.005	0.011	0.005
3. Acute renal failure	0.329	0.029	0.032	0.033	0.116
4. Adult respiratory distress syndrome	0.017	0.034	0.021	0.033	0.026
5. Amniotic fluid embolism	0.208	0.149	0.117	0.120	0.152
6. Cardiac arrest/ventricular fibrillation	0.022	0.014	0.032	0.033	0.025
7. Conversion of cardiac rhythm	0.000	0.000	0.005	0.011	0.004
8. Disseminated intravascular coagulation	0.126	0.197	0.197	0.169	0.170
9. Eclampsia	1.491	1.167	1.272	1.097	1.268
10. Heart failure/arrest during surgery or procedure*	NA	NA	NA	NA	NA
11. Puerperal cerebrovascular disorders	0.052	0.053	0.101	0.071	0.068
12. Pulmonary oedema/acute heart failure	0.260	0.216	0.240	0.229	0.237
13. Severe anaesthesia complications	0.009	0.005	0.000	0.027	0.010
14. Sepsis	0.629	0.908	1.176	1.075	0.928
15. Shock	1.066	0.888	0.931	0.993	0.972
16. Sickle cell disease with crisis	0.000	0.000	0.000	0.000	0.000
17. Air and thrombotic embolism	0.030	0.048	0.069	0.076	0.054
18. Blood products transfusion	32.254	24.824	30.865	29.792	29.465
19. Hysterectomy	0.746	0.879	0.825	0.742	0.798
20. Temporary tracheostomy	0.000	0.000	0.000	0.000	0.000
21. Ventilation	0.000	0.014	0.256	1.004	0.290
Number of delivery cases	230 700	208 263	187 880	183 206	810 049

There was no ICD-10 code in China that could indicate heart failure/arrest during surgery or procedure. Hence, the tenth indicator was not able to be identified in our data.

ICD-10, International Classification of Diseases Tenth Revision; NA, not available; SMM, severe maternal morbidity.

The first column of table 2 shows the descriptive statistics for the whole group. The overall SMM rate was about 3.31%, and the median maternal age was 27 years. Around 10.97% of mothers belonged to ethnic minority groups, 95.98% had been married and 50.69% were residents living in an urban area. The most popular insurance types of patients were NCMS (30.53%), URBMI (24.2%) and fully self-paid (21.85%). High-risk delivery accounted for 49.74%. Most patients were admitted through the outpatient department (77.92%), while nearly 20% were admitted through the emergency department. Regarding hospital levels and ownership types, most patients gave birth in tertiary and public non-profit institutions, while urban institutions had more deliveries than rural institutions. The total number of deliveries decreased between 2016 and 2019.

**Table 2:** Descriptive statistics

Variables n (%) or m (IQR)	Overall	Low volume (1–18)	Medium volume (19–142)	High volume (143–5664)	*P* value
SMM n (%)					
No	783 254 (96.69)	9379 (98.23)	95 436 (98.02)	678 439 (96.49)	<0.001 ***
Yes	26 795 (3.31)	169 (1.77)	1931 (1.98)	24 695 (3.51)	
Delivery volume m (IQR)	501 (260–893)	11 (6–14)	88 (19–114)	584 (346–962)	<0.001 ***
Patient characteristics					
Age m (IQR)	27 (24–30)	27 (23–30)	26 (23–30)	27 (24–30)	<0.001 ***
Minority n (%)					
No	721 177 (89.03)	8567 (89.73)	83 979 (86.25)	628 631 (89.40)	<0.001 ***
Yes	88 872 (10.97)	981 (10.27)	13 388 (13.75)	74 503 (10.60)	
Married n (%)					
No	32 551 (4.02)	423 (4.43)	4351 (4.47)	27 777 (3.95)	<0.001 ***
Yes	777 498 (95.98)	9125 (95.57)	930,16 (95.53)	675 357 (96.05)	
Living in urban/rural n (%)					
Rural	399 469 (49.31)	5861 (61.38)	59 572 (61.18)	334 035 (47.51)	<0.001 ***
Urban	410 581 (50.69)	3687 (38.62)	37 795 (38.82)	369 099 (52.49)	
Insurance type n (%)					
UEBMI	92 762 (11.45)	302 (3.16)	7942 (8.16)	84 518 (12.02)	<0.001 ***
URBMI	196 047 (24.20)	3840 (40.22)	29 804 (30.61)	162 403 (23.10)	
NCMS	247 315 (30.53)	2978 (31.19)	34 392 (35.32)	209 945 (29.86)	
Fully self-paid	176 960 (21.85)	1343 (14.07)	17 216 (17.68)	158 401 (22.53)	
Others	96 965 (11.97)	1085 (11.36)	8013 (8.23)	87 867 (12.5)	
High-risk delivery n (%)					
No	407 140 (50.26)	7417 (77.68)	61 019 (62.67)	338 704 (48.17)	<0.001 ***
Yes	402 909 (49.74)	2131 (22.32)	36 348 (37.33)	364 430 (51.83)	
Admission source n (%)					
Transferred from the emergency department within the hospital	156 324 (19.30)	1040 (10.89)	14 825 (15.23)	140 459 (19.98)	<0.001 ***
Transferred from the outpatient department within the hospital	631 184 (77.92)	7841 (82.12)	78 678 (80.81)	544 665 (77.46)	
Transferred from other hospital	2180 (0.27)	14 (0.15)	153 (0.16)	2013 (0.29)	
Others	20 361 (2.51)	653 (6.84)	3711 (3.81)	15 997 (2.28)	
Institutional characteristics					
Hospital level n (%)					
Tertiary	356 696 (44.03)	117 (1.23)	6981 (7.17)	349 598 (49.72)	<0.001 ***
Secondary	338 066 (41.73)	1094 (11.46)	42 418 (43.57)	294 554 (41.89)	
Primary or ungraded	115 287 (14.23)	8337 (87.32)	47 968 (49.27)	58 982 (8.39)	
Location n (%)					
Rural	351 502 (43.39)	5902 (61.81)	59 168 (60.77)	286 432 (40.74)	<0.001 ***
Urban	458 547 (56.61)	3646 (38.19)	38 199 (39.23)	416 702 (59.26)	
Ownership and profit n (%)					
Public non-profit	729 561 (90.06)	7548 (79.05)	78 255 (80.37)	643 758 (91.56)	<0.001 ***
Private non-profit	29 109 (3.59)	632 (6.62)	6305 (6.48)	22 172 (3.15)	
Private for profit	51 379 (6.34)	1368 (14.33)	12 807 (13.15)	37 204 (5.29)	
Number of beds m (IQR)	344 (129–800)	80 (50–102)	125 (70–250)	409 (150–944)	<0.001 ***
Number of beds for OG m (IQR)	53 (30–90)	10 (5–15)	20 (15–30)	60 (38–95)	<0.001 ***
Regional characteristics					
GDP per capita m (IQR)	37 589 (27 014–60 311)	31 651 (23 020–42 642)	33 666 (24 309–49 522)	39 033 (27 539–62 208)	<0.001 ***
Urbanisation rate m (IQR)	49.47 (39.03–71.75)	40.75 (36.56–50.55)	41.4 (36.81–53.31)	51.48 (39.71–72.41)	<0.001 ***
Year n (%)					
2016	230 700 (28.48)	2308 (24.17)	23 851 (24.5)	204 541 (29.09)	<0.001 ***
2017	208 263 (25.71)	2637 (27.62)	26 123 (26.83)	179 503 (25.53)	
2018	187 880 (23.19)	2414 (25.28)	24 561 (25.22)	160 905 (22.88)	
2019	183 206 (22.62)	2189 (22.93)	22 832 (23.45)	158 185 (22.5)	
Number of delivery cases	810 049	9548	97 367	703 134	

The categorical variables were described by number and percentage ‘n (%)’, the continuous variables were described by median and IQR ‘m (IQR)’.

χ^2^ tests were employed for the categorical variables, Kruskal-Wallis rank-sum tests were employed for the continuous variables.

Significant: ‘***’p<0.001, ‘**’ p<0.01, ‘*’ p<0.05.

GDP per capita, gross domestic product per capita; NCMS, New Cooperative Medical Scheme; OG, Obstetrics and Gynecology; SMM, severe maternal morbidity; UEBMI, Urban Employment Basic Medical Insurance; URBMI, Urban Residents Basic Medical Insurance.

The other columns in table 2 showed the differences between delivery volume groups. All the differences were tested by the χ^2^ test or Kruskal-Wallis rank-sum test. Due to the large sample size, all the differences were statistically significant. Without any adjustment, SMM was more frequent in the high-volume delivery institution than in the low-volume and medium-volume institutions. At the same time, the proportion of high-risk delivery in the high-volume institution (51.83%) was more than twice as much as the proportion in low-volume institutions (22.32%). The positive correlation suggested that patient heterogeneity was an important confounding factor that could cause endogenous problems. The proportion of patients belonging to ethnic minority groups was highest in the medium-volume institutions, while the proportion of married mothers was the highest in the high-volume institutions. In terms of health insurance types, the UEBMI and fully self-paid patients preferred high volume institutions, the URBMI patients preferred low-volume institutions and the NCMS patients preferred medium institutions. In addition, patients living in rural areas were more likely to choose low-volume and medium-volume institutions than in urban regions. The median for the number of beds, doctors and nurses, the regional GDP per capita and urbanisation rate increased with increased delivery volumes. The number of deliveries within each volume subgroup decreased from 2017 to 2019.

**Figure 2:**
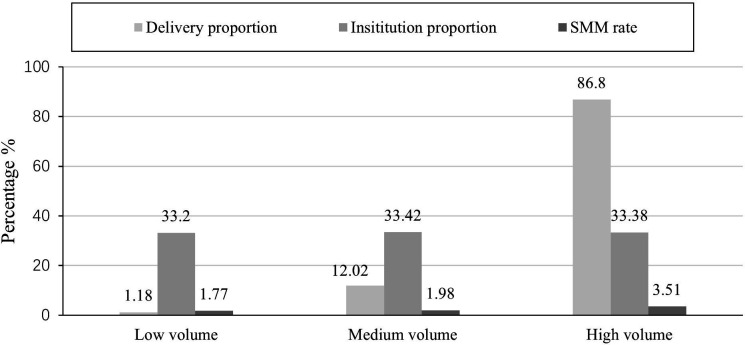
Delivery proportion, institution proportion and severe maternal morbidity (SMM) rate by delivery volume groups.

At the institutional level, the obstetric deliveries were mainly clustered in high-volume institutions. As illustrated by figure 2, the high-volume institutions provided more than 86% of delivery services, while the low-volume institutions only offered less than 2% of delivery services. The crude SMM rate without any adjustment was higher in the high-volume institutions than in the medium-volume and low-volume institutions (figure 2).

### Regression analysis

The quarterly delivery volume was divided by 1000 before being added into the regression models to make the results more readable. The VIF values were all below 10, suggesting that the multicollinearity levels could be accepted. Table 3 shows the estimation results of regression models (table 3).

**Table 3:** Marginal effects of estimates

Linear probability model	(1)	(2)	(3)	(4)	(5)
Delivery volume	0.003*** (0.003 to 0.004)	0.000 (0.000 to 0.000)	−0.025*** (−0.026 to –0.024)	0.003 (−0.003 to 0.009)	−0.021*** (−0.022 to –0.020)
Patient characteristics		√			√
Institutional characteristics			√		√
Regional characteristics				√	√
Year		√	√	√	√
**Logistic regression model**	**(6)**	**(7)**	**(8)**	**(9)**	**(10)**
Delivery volume	0.003*** (0.003 to 0.004)	0.001 (−0.001 to 0.001)	−0.021*** (−0.022 to –0.020)	0.001*** (0.001 to 0.002)	−0.015*** (−0.016 to –0.014)
Patient characteristics		√			√
Institutional characteristics			√		√
Regional characteristics				√	√
Year		√	√	√	√
**Ordinary IV model**	**(11)**	**(12)**	**(13)**	**(14)**	**(15)**
Delivery volume	−0.027*** (−0.029 to –0.026)	−0.054*** (−0.056 to –0.052)	−0.153*** (−0.158 to –0.148)	−0.091*** (−0.095 to –0.087)	−0.133*** (−0.139 to –0.127)
Patient characteristics		√			√
Institutional characteristics			√		√
Regional characteristics				√	√
Year		√	√	√	√
First-stage *F* statistic	48 654	39 989	27 459	16 590	20 966
First-stage adjusted *R* ^2^	0.129	0.155	0.732	0.293	0.747
Test for endogeneity (p value)	<0.001	<0.001	<0.001	<0.001	<0.001
**IV-logistic model**	**(16)**	**(17)**	**(18)**	**(19)**	**(20)**
Delivery volume	−0.032*** (−0.033 to –0.030)	−0.066*** (−0.068 to –0.063)	−0.198*** (−0.204 to –0.192)	−0.122*** (−0.126 to –0.117)	−0.162*** (−0.169 to –0.155)
Patient characteristics		√			√
Institutional characteristics			√		√
Regional characteristics				√	√
Year		√	√	√	√
First-stage *F* statistic	48 654	39 989	27 459	16 590	20 966
First-stage adjusted *R* ^2^	0.129	0.155	0.732	0.293	0.747
Test for endogeneity (*P* value)	<0.001	<0.001	<0.001	<0.001	<0.001

95% CIs in parentheses.

The average marginal effects of logistic regression models and IV-logistic models were reported.

Significant: ‘***’p<0.001, ‘**’ p<0.01, ‘*’ p<0.05.

IV, instrumental variable.

Models (1) to (5) showed the results of linear probability models. In model (1), the association between delivery volume and SMM without any adjustment was estimated. The patient characteristics, institutional characteristics and regional characteristics were separately added into models (2) to (4) and were all contained in model (5). The dummy variables of the year were controlled in models (2) to (5). The results of linear probability models were not consistent. The coefficients of delivery volume were positive in models (1), (2) and (4), but not statistically significant in model (2) and (4). Model (3) and model (5) showed the negative association between delivery volume and SMM. Similar covariates settings were applied in logistic regression models (6) to (10). The AME estimations of logistic regression models were very similar to linear probability models. The coefficient of delivery volume in linear probability full model (5) was −0.021 (95% CI −0.022 to –0.020), which indicated that each increase of 1000 cases in delivery volume was associated with a 2.1% reduction of SMM rate. The AME of delivery volume in the logistic regression model (10) was −0.015 (95% CI −0.016 to –0.014), suggesting that each increase of 1000 deliveries was associated with a 1.5% reduction of SMM rate. The adjusted OR (AOR) in model (10) was 0.615 (95% CI 0.594 to 0.636), which showed that each increase of 1000 deliveries was associated with a 38.5% reduction of odds for SMM. The results of linear probability models and logistic regression models only measured the association between delivery volume and SMM rather than a causal effect.

The causal effects were estimated by IV models as shown in models (11) to (20). The significant *F* statistics and adjusted *R^2^* of the first-stage regression implied that the instrument was not weak. The coefficient for surrounding delivery volume average was positive as we had expected, suggesting that an increased delivery volume average in the surrounding areas was associated with increased delivery cases actually occurring in a specific institution (online supplemental table 2). The coefficients of delivery volume in all IV models were negative and the AORs were all smaller than 1 with different settings of covariates, suggesting that delivery volume was an independent protective factor of SMM. Model (15) implied that an increase of 1000 deliveries could reduce 13.3% in SMM rates. The marginal effect of ordinary IV estimation in model (15) was a constant value that equalled the coefficient. For the IV-logistic regression model, the marginal effect decreased with increased delivery volume, which is more reasonable than a constant value. The AME and AOR of the preferred specification model (20) were respectively −0.162 (95% CI −0.169 to –0.155) and 0.005 (95% CI 0.004 to 0.006), which implied that 1000 deliveries could lead to the reduction of 16.2% and 99.5% in the rates and odds of SMM, respectively. The full estimation results were shown in supplemental tables (online supplemental table 3).

From a vertical perspective, the first column of table 3 presented the estimates without any covariates, the second column displayed the estimates after controlling individual characteristics and year, the third column showed the estimates after controlling institutional characteristics and year, the fourth column presented the estimates after controlling regional characteristics and year, and the last column contained the estimates after controlling all the confounders. Regardless of which type of confounders were added separately, the estimates were reduced compared with the original ones, and the use of the IV method would further reduce the estimates. Of the three categories of factors, institutional characteristics had the greatest impact on the estimates, and its results were closest to the results in the fifth column considering all confounders. Of the remaining two categories of factors, regional characteristics had a greater impact on estimates than individual characteristics.

### Sensitivity analysis

We used three different IV-logistic regression models to assess the robustness of the results. The first sensitivity analysis replaced the 2 hours’ driving distance with 1 hour. The AME and AOR and their CIs were −0.153 (95% CI −0.159 to –0.147) and 0.007 (95% CI 0.006 to 0.008), respectively. Blood production transfusions were excluded from the second sensitivity analysis for analysing the dependent variable SMM. The AME and AOR and their CIs were respectively −0.162 (95% CI −0.169 to –0.155) and 0.294 (95% CI 0.183 to 0.470). The institutions with less than 10 deliveries per quarter were excluded from the third sensitivity analysis. A total of 4054 delivery cases and 1104 institution-years were excluded. The AME and AOR and their CIs turned out to be −0.163 (95% CI −0.170 to –0.156) and 0.005 (95% CI 0.004 to 0.006), respectively. These sensitivity analyses demonstrated the robust causal effect of delivery volume on SMM (online supplemental table 4).

## Discussions

Our study examined the protective causal effect of delivery volume on SMM. The adjusted marginal effect estimates of the linear probability model and the logistic regression model were much smaller than the IV estimates due to the endogenous problems. The institutional characteristics posed the largest impacts on the effect estimates, suggesting that such volume-outcome was the most apparent among healthcare institutions with similar characteristics. Two potential mechanisms might be used to explain such causal effect. Specifically, the ‘practice-makes-perfect’ learning effect serves as a contributor to the causal effect, meaning that doctors tend to acquire more medical expertise and gain more clinical experiences from an increased number of disease cases.^9^ In addition, large-scale institutions are typically composed of a wide range of clinical departments with high-quality medical resources, thus are capable of providing high-quality health services, which is the ‘economies of scale’ effect.^36^ Both mechanisms tend to pose potential impacts at the institutional level, thus partially contributing to the outcomes.

Our findings are consistent with the results of previous studies, including studies conducted by Bozzuto *et al* (2019),^11^ Aoyama *et al* (2019)^12^ and Campbell *et al* (2018).^13^ Such volume-outcome association has also been identified between delivery volume and caesarean delivery,^15 40–43^ delivery volume and postpartum haemorrhage,^14 44^ as well as between delivery volume and neonatal morbidity.^13 45^ The logistic regression model was the most commonly adopted model by previous studies as mentioned above. The hierarchical generalised linear model, marginal log-linear models, mixed-effects spline regression have also been applied by relevant studies, while none of them have investigated the causal effect or endogenous problems.

Our findings are different from the results reported by Booker *et al* (2018) and Clapp *et al* (2017), which suggested that delivery volume was not significantly associated with severe morbidity risk or caesarean section after controlling the patient and hospital characteristics.^16 18^ These two studies both employed multivariable regression models to measure the association instead of the causal effect. Besides, they both only used categorical variables to measure institutional heterogeneity, which was crude and insufficient. More accurate indicators reflective of institutional characteristics should be added to the models in order to avoid confounding problems.

All the relevant studies mentioned above are from the USA except two from Korea.^15 40^ To the best of our knowledge, this is the first study that provides evidence from a developing country in terms of the causal effect of delivery volume on maternal health outcome, which also sheds lights on China’s remarkable milestones on maternal health promotion from a novel perspective. In China, maternal mortality has been significantly reduced by dramatically increased in-hospital delivery rates over the past decades.^46–48^ In 2000, China initiated a safe motherhood programme that encouraged in-hospital delivery and discouraged community midwifery and home-based delivery.^49^ This policy promoted the primary centralisation of obstetric delivery services from families to healthcare institutions. The centralisation trend is still keeping on. As the nationwide fertility rate continues to decline, some low-volume delivery institutions may even have no cases of obstetric delivery like the other low-fertility countries.^19 50–53^ Meanwhile, the constantly intensified urbanisation as well as the labour force migration have also exacerbated the tendency of childbearing-age women to concentrate in urban areas. In Sichuan, the number of OG doctors rose from 14 244 in 2016 to 15 915 in 2019, while the number of delivery institutions decreased from 1168 to 1063 during the same period of time. Given the inevitable trend towards centralisation of delivery services, it is important and urgent to study the impact of centralisation on service quality. The causal effect of volume-outcome is a recognised and important approach to the impact of centralised strategy on service quality. It is of great significance to verify its existence in the field of delivery services in China. The results can provide a reference for policy makers to promote a centralised strategy.

We also took the lead in applying the SMM definition on hospital discharge data under the context of China’s healthcare system. As indicated by a recently published scoping review of SMM, diverse versions of SMM definitions have been applied.^27^ The SMM definition CDC basis has been mainly applied in the USA and further modified into the Canadian, Australian and Swedish versions.^27^ Likewise, this study adapted the SMM definition from the USA into a Chinese version. The ICD code-based SMM definition is a labour-saving and easily generalised method, which has great potential to be used for the long-term surveillance of obstetric quality. Meanwhile, SMM was sensitive to age-related risk.^27^ According to a previous study, 78.7% of maternal deaths were identified with SMM, and 1% of women with SMM died.^21^ Targeting on reducing SMM can make the death-related critical points controlled at an earlier stage and achieve early prevention for pregnant women.

It is difficult to recommend specific minimum delivery volume thresholds due to several study limitations. First, the discharge data used in our study only contained information collected during hospitalisations. SMM is only a relatively general maternal outcome indicator that partially reflects the quality of obstetric delivery. More specific indicators associated with both maternal and neonatal outcomes should be considered in the process of identifying delivery volume thresholds. Second, the SMM rate might have been underestimated in our study as the data sets we used for analysis only contained the first procedure codes, thus leading to the omission of some SMM cases. As an attempt to identify the thresholds, we divided the delivery volume into two groups and estimated the coefficients of the high-volume delivery group in models. The ordinary IV models and TSLS estimation were applied. The distribution of delivery volume showed a skewness distribution (online supplemental figure 1), while the 25%, 50% and 75% quantiles of quarterly delivery volume at the individual level were 260, 501 and 893, respectively. Therefore, the threshold value was set up as an integer ranging from 10 to 1000. The coefficients and the significance changed accordingly with changed threshold values as described in online supplemental figure 2. The *P* values for the coefficient test were far below 0.001. The coefficients were all negative and their absolute values decreased with increased values of thresholds. As indicated by the figure, the marginal effect of delivery volume became significantly smaller after the threshold value was set up to 200.

Under the current trends of obstetric services towards centralisation, ensuring the accessibility of obstetric services has become a critical challenge in China. On the other hand, spatial accessibility is rather essential for maintaining the in-hospital delivery rate at a relatively high level.^54 55^ As such a centralisation trend is very much likely to induce a reduction in in-hospital delivery rates as well as an increase in maternal mortality, the centralisation-facilitated maternal health promotion at the potential sacrifice of spatial accessibility requires close attention.

## Conclusion

Through the adoption of IV estimation, our study demonstrated the causal effect of delivery volume on SMM. As suggested by IV-logistic estimation, each increase of 1000 cases in the delivery volume would lead to the reduction of 16.2% and 99.5% in the rates and odds of SMM, respectively. From a long-term perspective, centralisation of obstetric delivery services is very much likely to result in an increased number of high-volume healthcare institutions as well as improved maternal health outcomes. Under such context, the impact of such centralisation on pregnant women’s accessibility of medical services requires close attention.

## Data Availability

The data that support the findings of this study are not publicly available due to privacy or ethical restrictions. They are part of the health administrative data sets of Sichuan, China.
