# Supplementary material for: The causal effect of delivery volume on severe maternal morbidity: an instrumental variable analysis in Sichuan, China
Source: BMJ Glob Health. 2022 May 10;7(5):e008428. doi: 10.1136/bmjgh-2022-008428 (PMC9092146; doi:10.1136/bmjgh-2022-008428)
Supplement: Supplementary data [file bmjgh-2022-008428supp001.pdf]

Supplemental Table 1 Identification codes for high-risk delivery and severe maternal morbidity

Identification objectives	Indicators	ICD-10 codes/ ICD-9-CM codes
**Severe maternal morbidity**	1. Acute myocardial infarction	I21, I22
	2. Aneurysm	I71, I79.0
	3. Acute renal failure	N17
	4. Adult respiratory distress syndrome	J80, J95.1, J95.2, J95.3, J96.0, R09.2
	5. Amniotic fluid embolism	O88.1
	6. Cardiac arrest/ventricular fibrillation	I46, I49.0
	7. Conversion of cardiac rhythm	99.6
	8. Disseminated intravascular coagulation	D65, D68.8, D68.9, O72.3
	9. Eclampsia	O15
	10. Heart failure/arrest during surgery or procedure	-
	11. Puerperal cerebrovascular disorders	I60, I61, I62, I63, I64, I65, I66, I67, I68, O22.5, O87.3
	12. Pulmonary edema / Acute heart failure	J81, I50.1, I50.0, I50.9
	13. Severe anesthesia complications	O74.0, O74.1, O74.2, O74.3, O89.0, O89.1, O89.2
	14. Sepsis	O85, O86.0, T80.2, T81.411, A40, A41, A32.7
	15. Shock	O75.1, R57, T78.2, T88.2, T88.6, T81.102
	16. Sickle cell disease with crisis	D57.0
	17. Air and thrombotic embolism	I26 O88.0, O88.2, O88.3, O88.8
	18. Blood products transfusion	99.0
	19. Hysterectomy	68.3, 68.4, 68.5, 68.6, 68.7, 68.8, 68.9
	20. Temporary tracheostomy	31.1
	21.Ventilation	93.9, 96.02, 96.03, 96.05
**High-risk delivery**	Risk codes from the SMFM definition	B20, B21, B22, B23, B24, N85.801, O00.807, O10.2, O10.3, O14.101, O22.5, O24, O30, O31, O32.1, O32.2, O32.3, O33.6, O33.7, O34.2, O35.0, O36.4, O36.502, O43.1, O43.8, O43.9, O44, O45, O60.0, O60.1, O64, O66.1, O66.2, O66.401, O66.5, O69.0, O69.4, O71.0, O71.1,

		O73, O75.7, O98.7 , O99.4, Z21, Z37.1, Z37.2, Z37.3, Z37.4, Z37.5, Z37.6, Z37.7, Z86.32, Z87.74
	Risk codes from the Chinese expert's consensus	C53, D06, N85.801, O00.807, O14.1, O14.101, O15.0, O15.1, O15.9, O22.1, O26.606 , O26.607, O30, O31, O32.1, O32.2, O32.3, O32.500, O33.5, O34.2, O35.811, O36.3, O36.6, O43.001, O44, O45, O60.1, O60.3, O64, O66.1, O66.2, O66.401, O68, O69.0, O69.209, O69.4, O75.7, O84, O98.2, O98.302 , O99.101, O99.102, O99.4, Z43.7, Q52.104, Z37.2, Z37.3, Z37.4, Z37.5, Z37.6, Z37.7, Z38.3, Z38.4, Z38.5, Z38.6, Z38.7, Z38.8

Notes:

1. There is no ICD-10 code in China that could indicate heart failure/arrest during surgery or procedure, so the tenth indicator could not be identified with the discharge data in China.

2. For enhancing the identification for the blood products transfusion, the cases with fees for the blood products in the discharge record were considered to already have the blood products transfusion.

Supplemental Table 2 The first-stage results of IV estimation

Variables	Coefficient (95% CI)	*P* value
**Instrumental variable**	0.067 (0.066,0.068)	<0.001 ***
**Age**	-0.026 (-0.028,-0.024)	<0.001 ***
**Minority**		
No	1 [Reference]	
Yes	1.569 (1.541,1.597)	<0.001 ***
**Unmarried**		
No	1 [Reference]	
Yes	0.325 (0.283,0.368)	<0.001 ***
**Living in urban/rural**		
Urban	1 [Reference]	
Rural	0.453 (0.427,0.479)	<0.001 ***
**Insurance type**		
UEBMI	1 [Reference]	
URBMI	0.042 (0.011,0.072)	0.007**
NCMS	-0.241 (-0.271,-0.210)	<0.001 ***
Fully self-paid	0.012 (-0.018,0.042)	0.430
Others	-1.509 (-1.544,-1.475)	<0.001 ***
**High-risk delivery**		
No	1 [Reference]	
Yes	-0.283 (-0.301,-0.266)	<0.001 ***
**Admission source**		
Transferred from the emergency department within the hospital	1 [Reference]	
Transferred from the outpatient department within the hospital	0.322 (0.301,0.342)	<0.001 ***
Transferred from other hospital	-0.208 (-0.366,-0.050)	0.010**
Others	1.159 (1.103,1.215)	<0.001 ***
**Hospital level**		
Primary or ungraded	-1.287 (-1.314,-1.261)	<0.001 ***
Secondary	1 [Reference]	
Tertiary	1.310 (1.287,1.333)	<0.001 ***
**Location**		
Urban	1 [Reference]	
Rural	0.351 (0.318,0.383)	<0.001 ***
**Ownership and profit**		
Public nonprofit	1 [Reference]	
Private nonprofit	0.363 (0.317,0.410)	<0.001 ***
Private for profit	0.430 (0.393,0.468)	<0.001 ***
**Number of beds**	-0.001 (-0.001,-0.001)	<0.001 ***
**Number of beds for OG**	0.064 (0.064,0.064)	<0.001 ***
**GDP per capita**	0.029 (0.028,0.029)	<0.001 ***

**Urbanization rate**	-0.020 (-0.020,-0.019)	<0.001 ***
**Year**		
2016	1 [Reference]	
2017	-1.556 (-1.579,-1.533)	<0.001 ***
2018	-2.438 (-2.463,-2.414)	<0.001 ***
2019	-2.387 (-2.413,-2.361)	<0.001 ***

Notes:

1. Instrumental variable was the average number of deliveries per institution in the surrounding region for a specific delivery institution.
2. UEBMI, Urban Employment Basic Medical Insurance; URBMI, Urban Residents Basic Medical Insurance; NCMS, New Cooperative Medical Scheme; GDP per capita, Gross Domestic Product per capita.
3. 95% confidence intervals in parentheses.
4. Significant: '***'*P*<0.001, '**' *P*<0.01, '*' *P* <0.05.

BMJ Publishing Group Limited (BMJ) disclaims all liability and responsibility arising from any reliance placed on this supplemental material which has been supplied by the author(s)

Supplemental Table 3 Marginal effects of linear probability model, logistic regression model, ordinary IV model and IV-logistic model estimates (full models)

Variable	Linear probability model (5)	Logistic regression model (10)	Ordinary IV model (15)	IV-logistic model (20)
**Delivery volume**	-0.021*** (-0.022,-0.020)	-0.015*** (-0.016,-0.014)	-0.133*** (-0.139,-0.127)	-0.162*** (-0.169,-0.155)
**Age**	0.001*** (0.000,0.001)	0.001*** (0.000,0.001)	0.000*** (0.000,0.000)	0.000 (0.000,0.000)
**Minority**				
No	1 [Reference]	1 [Reference]	1 [Reference]	1 [Reference]
Yes	0.016*** (0.015,0.017)	0.020*** (0.018,0.022)	0.034*** (0.033,0.036)	0.062*** (0.058,0.066)
**Unmarried**				
No	1 [Reference]	1 [Reference]	1 [Reference]	1 [Reference]
Yes	-0.001 (-0.003,0.001)	-0.001 (-0.003,0.001)	0.002 (-0.000,0.004)	0.003** (0.001,0.006)
**Living in urban/rural**				
Urban	1 [Reference]	1 [Reference]	1 [Reference]	1 [Reference]
Rural	0.010*** (0.009,0.011)	0.006*** (0.005,0.007)	0.013*** (0.011,0.014)	0.011*** (0.010,0.012)
**Insurance type**				
UEBMI	1 [Reference]	1 [Reference]	1 [Reference]	1 [Reference]
URBMI	-0.004*** (-0.006,-0.003)	-0.001 (-0.003,0.000)	-0.004*** (-0.006,-0.003)	-0.002** (-0.004,-0.001)
NCMS	0.001	0.005***	-0.004***	-0.002*

	(-0.000,0.003)	(0.004,0.006)	(-0.005,-0.002)	(-0.003,-0.000)
Fully self-paid	0.003*** (0.002,0.005)	0.007*** (0.006,0.008)	0.004*** (0.002,0.005)	0.007*** (0.005,0.008)
Others	-0.010*** (-0.011,-0.008)	-0.005*** (-0.007,-0.004)	-0.025*** (-0.027,-0.023)	-0.021*** (-0.022,-0.019)
**High-risk delivery**				
No	1 [Reference]	1 [Reference]	1 [Reference]	1 [Reference]
Yes	0.012*** (0.011,0.013)	0.012*** (0.011,0.012)	0.009*** (0.008,0.010)	0.008*** (0.007,0.009)
**Admission source**				
Transferred from the emergency department within the hospital	1 [Reference]	1 [Reference]	1 [Reference]	1 [Reference]
Transferred from the outpatient department within the hospital	-0.004*** (-0.005,-0.003)	-0.003*** (-0.004,-0.002)	0.000 (-0.001,0.001)	0.002*** (0.001,0.003)
Transferred from other hospital	0.022*** (0.014,0.029)	0.003 (-0.001,0.009)	0.018*** (0.010,0.026)	-0.002 (-0.006,0.002)
Others	-0.017*** (-0.020,-0.014)	-0.020*** (-0.022,-0.018)	-0.006*** (-0.009,-0.004)	-0.009*** (-0.012,-0.006)
**Hospital level**				
Primary or ungraded	1 [Reference]	1 [Reference]	1 [Reference]	1 [Reference]
Secondary	0.007*** (0.006,0.008)	0.012*** (0.011,0.012)	0.023*** (0.021,0.024)	0.016*** (0.015,0.016)
Tertiary	0.016*** (0.015,0.018)	0.029*** (0.028,0.031)	0.048*** (0.046,0.050)	0.060*** (0.058,0.063)
**Location**				
Urban	1 [Reference]	1 [Reference]	1 [Reference]	1 [Reference]

Rural	-0.020***<br>(-0.021,-0.018)	-0.018***<br>(-0.019,-0.016)	-0.016***<br>(-0.017,-0.014)	-0.012***<br>(-0.014,-0.011)
**Ownership and profit**				
Public nonprofit	1 [Reference]	1 [Reference]	1 [Reference]	1 [Reference]
Private nonprofit	0.015***<br>(0.013,0.017)	0.022***<br>(0.018,0.025)	0.021***<br>(0.019,0.023)	0.042***<br>(0.037,0.047)
Private for profit	0.025***<br>(0.023,0.026)	0.049***<br>(0.045,0.053)	0.030***<br>(0.028,0.032)	0.074***<br>(0.069,0.079)
**Number of beds**	0.000***<br>(0.000,0.000)	0.000***<br>(0.000,0.000)	0.000***<br>(0.000,0.000)	0.000***<br>(0.000,0.000)
**Number of beds for OG**	0.000***<br>(0.000,0.000)	0.000***<br>(0.000,0.000)	0.001***<br>(0.001,0.001)	0.001***<br>(0.001,0.001)
**GDP per capita**	-0.001***<br>(-0.001,-0.001)	-0.001***<br>(-0.001,-0.001)	-0.000***<br>(-0.000,-0.000)	-0.000***<br>(-0.000,-0.000)
**Urbanization rate**	0.001***<br>(0.001,0.001)	0.001***<br>(0.001,0.001)	0.000***<br>(0.000,0.000)	0.000***<br>(0.000,0.000)
**Year**				
2016	1 [Reference]	1 [Reference]	1 [Reference]	1 [Reference]
2017	-0.011***<br>(-0.012,-0.010)	-0.011***<br>(-0.012,-0.010)	-0.034***<br>(-0.035,-0.032)	-0.059***<br>(-0.062,-0.055)
2018	-0.007***<br>(-0.008,-0.006)	-0.006***<br>(-0.005,-0.005)	-0.040***<br>(-0.042,-0.038)	-0.066***<br>(-0.070,-0.062)
2019	-0.006***<br>(-0.007,-0.004)	-0.004***<br>(-0.005,-0.002)	-0.039***<br>(-0.041,-0.037)	-0.065***<br>(-0.069,-0.061)

Notes:

1. UEBMI, Urban Employment Basic Medical Insurance; URBMI, Urban Residents Basic Medical Insurance; NCMS, New Cooperative Medical Scheme; GDP per capita, Gross Domestic Product per capita.
2. 95% confidence intervals in parentheses.
3. Significant: '***' P<0.001, '**' P<0.01, '*' P <0.05.

Supplemental Table 4 Sensitivity analysis by IV-logistic regression models

Variable	Model 1 (One-hour driving distance)	Model 2 (Blood transfusions were excluded)	Model 3 (≥10 deliveries)
**Delivery volume**	-0.153*** (-0.159,-0.147)	-0.162*** (-0.169,-0.155)	-0.163*** (-0.170,-0.156)
**Age**	0.000* (0.000,0.000)	0.000 (0.000,0.000)	0.000 (0.000,0.000)
**Minority**			
No	1 [Reference]	1 [Reference]	1 [Reference]
Yes	0.060*** (0.056,0.064)	0.062*** (0.058,0.066)	0.062*** (0.059,0.066)
**Unmarried**			
No	1 [Reference]	1 [Reference]	1 [Reference]
Yes	0.003* (0.000,0.005)	0.003** (0.001,0.006)	0.003* (0.001,0.006)
**Living in urban/rural**			
Urban			
Rural	0.011*** (0.010,0.012)	0.011*** (0.010,0.012)	0.011*** (0.010,0.013)
**Insurance type**			
UEBMI	1 [Reference]	1 [Reference]	1 [Reference]
URBMI	0.000 (-0.002,0.001)	-0.002** (-0.004,-0.001)	-0.002** (-0.004,-0.001)
NCMS	0.001	-0.002*	-0.002*

	(-0.001,0.002)	(-0.003,0.000)	(-0.003,0.000)
Fully self-paid	0.009***<br>(0.008,0.011)	0.007***<br>(0.005,0.008)	0.007***<br>(0.005,0.009)
Others	-0.018***<br>(-0.019,-0.017)	-0.021***<br>(-0.022,-0.019)	-0.021***<br>(-0.022,-0.020)
**High-risk delivery**			
No	1 [Reference]	1 [Reference]	1 [Reference]
Yes	0.009***<br>(0.008,0.009)	0.008***<br>(0.008,0.009)	0.008***<br>(0.008,0.009)
**Admission source**			
Transferred from the emergency department within the hospital	1 [Reference]	1 [Reference]	1 [Reference]
Transferred from the outpatient department within the hospital	0.002***<br>(0.001,0.003)	0.002***<br>(0.001,0.003)	0.002***<br>(0.001,0.003)
Transferred from other hospital	-0.003<br>(-0.007,0.002)	-0.002<br>(-0.006,0.002)	-0.002<br>(-0.006,0.002)
Others	-0.010***<br>(-0.012,-0.007)	-0.009***<br>(-0.012,-0.006)	-0.009***<br>(-0.012,-0.006)
**Hospital level**			
Primary or ungraded	1 [Reference]	1 [Reference]	1 [Reference]
Secondary	0.016***<br>(0.015,0.016)	0.016***<br>(0.016,0.017)	0.016***<br>(0.015,0.017)
Tertiary	0.057***<br>(0.055,0.059)	0.061***<br>(0.058,0.063)	0.061***<br>(0.059,0.063)
**Location**			
Urban	1 [Reference]	1 [Reference]	1 [Reference]

Rural	-0.013***<br>(-0.014,-0.011)	-0.013***<br>(-0.014,-0.011)	-0.013***<br>(-0.015,-0.011)
**Ownership and profit**			
Public nonprofit	1 [Reference]	1 [Reference]	1 [Reference]
Private nonprofit	0.039***<br>(0.035,0.044)	0.043***<br>(0.038,0.047)	0.044***<br>(0.039,0.049)
Private for profit	0.068***<br>(0.064,0.073)	0.074***<br>(0.069,0.079)	0.076***<br>(0.071,0.081)
**Number of beds**	0.000<br>(0.000,0.000)	0.000*<br>(0.000,0.000)	0.000*<br>(0.000,0.000)
**Number of beds for OG**	0.001***<br>(0.001,0.001)	0.001***<br>(0.001,0.001)	0.001***<br>(0.001,0.001)
**GDP per capita**	0.000***<br>(0.000,0.000)	0.000***<br>(0.000,0.000)	0.000***<br>(0.000,0.000)
**Urbanization rate**	0.000***<br>(0.000,0.000)	0.000***<br>(0.000,0.000)	0.000***<br>(0.000,0.000)
**Year**			
2016	1 [Reference]	1 [Reference]	1 [Reference]
2017	-0.056***<br>(-0.059,-0.052)	-0.059***<br>(-0.062,-0.055)	-0.059***<br>(-0.063,-0.056)
2018	-0.063***<br>(-0.066,-0.059)	-0.066***<br>(-0.070,-0.062)	-0.067***<br>(-0.071,-0.063)
2019	-0.061***<br>(-0.065,-0.057)	-0.065***<br>(-0.069,-0.061)	-0.066***<br>(-0.070,-0.062)

Notes:

1. UEBMI, Urban Employment Basic Medical Insurance; URBMI, Urban Residents Basic Medical Insurance; NCMS, New Cooperative

Medical Scheme; GDP per capita, Gross Domestic Product per capita.

2. Average marginal effects and 95% confidence intervals were reported.

3. Significant: ‘***’*P*<0.001, ‘**’ *P*<0.01, ‘*’ *P* <0.05.

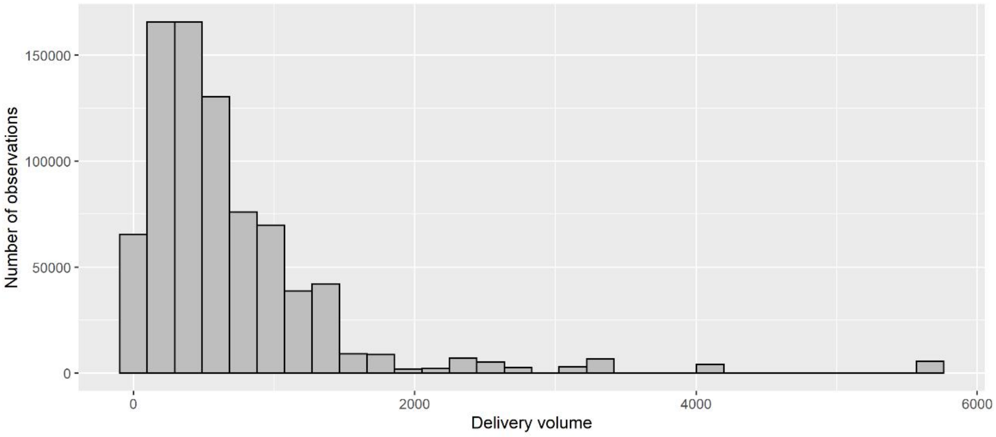

Supplemental Figure 1 The distribution of delivery volume

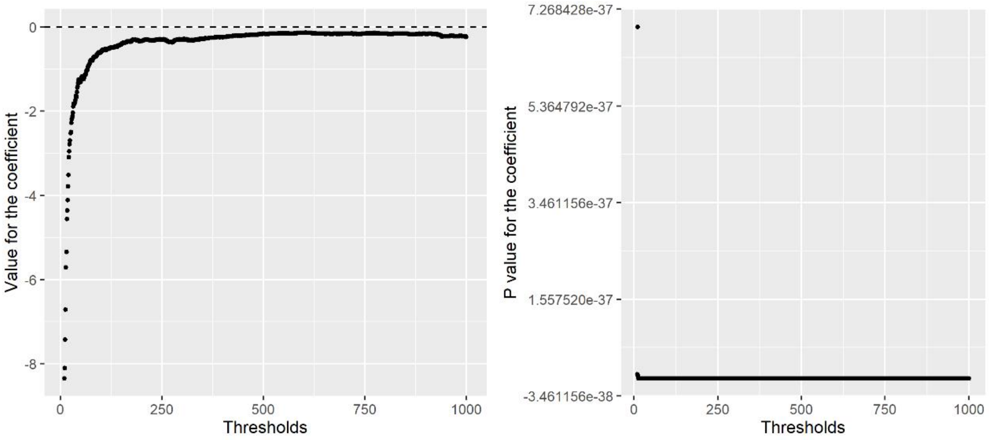

Supplemental Figure 2 The coefficients of the high-volume variable in ordinary IV models
